# Sporadic renal cell carcinoma in young and elderly patients: are there different clinicopathological features and disease specific survival rates?

**DOI:** 10.1186/1477-7819-5-16

**Published:** 2007-02-05

**Authors:** Stefan Denzinger, Wolfgang Otto, Maximilian Burger, Christine Hammerschmied, Kerstin Junker, Arndt Hartmann, Wolf F Wieland, Bernhard Walter

**Affiliations:** 1 Department of Urology, University of Regensburg, Regensburg, Germany; 2 Department of Urology, University of Jena, Germany; 3 Institute of Pathology, University of Regensburg, Regensburg, Germany

## Abstract

**Background:**

Sporadic renal cell carcinoma (RCC) is rare in young adults. In the present retrospective study we reviewed clinicopathological features and disease specific survival rates in young patients (≤45 years) with RCC and compared them to old patients (≥75 years) with RCC.

**Methods:**

Between 1992 and 2005 a total of 1042 patients were treated for RCC at our institution. We found 70 patients 45 years or younger (YP) and 150 patients 75 years or older (OP) at time of diagnosis. There were no differences in therapeutical approaches between both groups. Clinical and biologic parameters at diagnosis were compared and subjected to uni- and multivariate analysis to study cancer specific survival and progression rate. Mean postoperative follow-up in both groups was 50.1 months.

**Results:**

Mean age was 39 years in YP and 80 years in OP, respectively. YP demonstrated significantly lower stage (pT1-pT2 N0 M0, p = 0.03), lower tumor grade (p = 0.01) and higher male-to-female ratio (p < 0.001). The rate of lymph node metastases or distant metastatic disease at presentation did not differ significantly between both groups. In multivariate analysis young age was independently associated with a higher 5-year cancer specific survival (95.2% vs. 72.3%, p = 0.009) and a lower 5-year progression rate (11.3% vs. 42.5%, p = 0.002).

**Conclusion:**

Sporadic RCC in young patients have lower tumor stages and grades and a better outcome compared to elderly. Age≤45 years was an independent prognostic factor for survival and progression.

## Background

Renal cell carcinoma (RCC) is the most common renal parenchymal malignancy and represents 3% – 6% of all adult malignancies [1]. Patients are generally older than 40 years at diagnosis and the disease occurs predominantly in the seventh decade of life [2]. Only 3 to 7% of all sporadic RCC patients have been reported to be below 40 years [3-7].

In other tumor entities it has been noted that younger adults often have a less favorable prognosis than elderly patients. Solid organ malignancies including those of breast, colorectal and prostate in young patients have been already investigated [8-11] and these tumors seem to have a more pejorative survival prognosis. There have been anecdotal reports that renal masses may behave in a more biologically aggressive manner with poorer outcomes in younger patients [3,6]. To date, the rare studies on early onset RCC have been mostly descriptive with a small number of patients and a relatively short follow-up (table 1) [3,5,12-19]. Some studies lack a group of regular onset patients for comparison at all [12,13]. Many data are conflicting however and study designs vary considerably as well. In analogy to these conflicting data, major differences in methodology are found in the data published to date. In the present retrospective study we compare clinicopathological features and cancer specific survival of patients with confirmed RCC aged 45 years or younger with patients 75 years or older in univariate and multivariate analysis. By setting a minimum of 30 years between the groups we hoped to increase the discrimination and to shed further light on the clinical behavior of young versus old patients with RCC.

**Table 1:** Previous RCC studies and cancer specific survival rates of young and old RCC patients.

**Study**	**Study period**	**Young group**		**Older group**		**5-year cancer specific survival rate**		**p Value**
		**No. patients**	**Mean age**	**No. patients**	**Mean age**	**Young**	**Old**	

Boykin et al. [3]	1947–1989	14		***	***	70%	***	
Abou El Fettouh et al. [12]	1981–2001	101	33.7 y	***	***	67%	***	
Eggener et al. [13]	1988–2002	91	37.1 y	***	***	88%	***	
Schiff et al. [5]	1948–1980	37	31.0 y	486 (≥41 y)	***	92%*	45%*	p < 0.01
Rainwater et al. [14]	1970–1986	41	35.7 y	34 (≥80 y)	82.4 y	74%	70%	n.s.
Yusim et al. [14]	1985–1997	15	37.0 y	107 (≥50 y)	63.0 y	93%	77%	p < 0.05
Gillett et al. [16]	1970–2000	107	***	958 (60–70 y)	***	75%**	72%**	n.s.
Goetzl et al. [17]	1989–2002	34	35.0 y	99 (≥41 y)	65.0 y	85%	84%	n.s.
Sanchez-Ortiz et al. [18]	1992–2002	106	34.7 y	145 (58–61 y)	59.4 y	66%	52%	p < 0.01
Siemer et al. [19]	1975–2004	87	34.1 y	2164 (≥41 y)	***	78%	72%	n.s.

* only of stage I RCC

** only of clear cell RCC

*** no data available

y = years

n.s. = not significant (p < 0.05)

## Methods

Between 1992 and 2005 a total of 1042 patients underwent partial or radical nephrectomy for renal masses at the Department of Urology of the University of Regensburg. The medical and histopathological data of 70 YP (range: 26 to 45 years, mean: 39 years) and 150 OP (range: 75 to 91 years, mean: 80 years) suffering from RCC were reviewed We did not include middle-aged patients (>45 years and < 75 years) to avoid masking any potential differences in tumor biology between young and old patients. All familial RCC syndromes including von Hippel-Lindau disease were excluded. Pathological evaluation was reviewed and tumor stage was adapted according to the 2002 TNM classification. Patients were evaluated preoperatively by physical examination, laboratory studies, ultrasonography and radiographic staging including chest x-ray and computerized tomography. Staging lymph nodes dissection was performed for all patients. The postoperative outcomes and duration of follow-up were compiled by chart review and patient interview. Complete follow-up information was available for all patients through a department registry. Statistical analysis were completed using SPSS version 12.0 (SPSS, Chicago, IL). Contingency table analysis and two-sided Fisher's exact tests were used to study the statistical association between the two groups. Significant differences concerning tumor progression and cancer specific survival were calculated using the Kaplan-Meier method and log rank test. For cancer related death, patients were surveyed at the time of their last clinical follow-up appointment or at their date of death not related to the tumor. Deaths from causes other than RCC were censored. Uni- and multivariate Cox proportional hazards models were performed to evaluate the relationship between cancer specific survival, tumor progression, TNM pathologic stage, histologic subtypes, tumor grade, incidental or symptomatic disease and, in addition to other variables, age (separated into YP and OP). P values < 0.05 were considered significant.

## Results

### Comparison of histopathological and clinical parameters according to age

#### Epidemiology

Of the 86 young patients undergoing renal surgery, 70 (81.3%) were treated for RCC, 15 (17.5%) for benign lesions like oncocytoma and angiomyolipoma and one (1.2%) for lymphoma. Within consecutively chosen 179 older patients RCC dominated with 150 (83.8%) cases, benign renal masses were found in 28 (15.6%) patients and leiomyosarcoma in one patient (0.6%). While the expected sex ratio was seen in YP with 76% male patients, it was reversed in OP (43%; p < 0.001) (table 2).

**Table 2:** Comparison of clinical and pathological features of young (YP) and elderly (OP) RCC patients.

	YP (70 patients) No. (%)	OP (150 patients) No. (%)	p Value
Sex			**< 0.001**
Female	17 (24%)	86 (57%)	
Male	53 (76%)	64 (43%)	
Sex ratio (male/female)	3.1	0.7	
			
Clinical presentation			p = 0.19
Symptomatic	20 (28.4%)	52 (34,5%)	
Asymptomatic	50 (71.6%)	98 (65.5%)	
			
WHO 2002 stage of primary tumor			**p = 0.03**
pT1- pT2, N0, M0	60 (85.7%)	101 (67.3%)	
pT3, N0, M0	7 (10.0%)	29 (19.3%)	
pT4 and/or N+, and/or M+	3 (4.3%)	20 (13.4%)	
			
Tumor grade			**p = 0.01**
Grade 1	24 (34,3%)	24 (16.0%)	
Grade 2	35 (50.0%)	110 (73.3%)	
Grade 3	11 (15.7%)	16 (10.7%)	
			
RCC subtypes:			p = 0.32
Clearcell RCC	47 (67.2%)	98 (65.3%)	
Papillary RCC	13 (18.6%)	29 (19.4%)	
Chromophobe RCC	5 (7.1%)	16 (10.7%)	
Other histological types	5 (7.1%)	7 (4.6%)	

bold face representing p-values < 0.05

RCC = renal cell carcinoma

#### Etiology

YP were more likely to smoke tobacco (>10 cigarettes per day, 46.3% vs. 9.7%, p < 0.001) but did not differ concerning obesity (BMI >30; 20.9% vs. 22.1%, p = 0.93). There were no significant differences in the rate of secondary malignancies (p = 0.07).

#### Clinical presentation

RCC was an incidental finding in 71.6% YP and 65.5% OP (p = 0.19). In both groups dominant symptoms were flank pain (17.9% vs. 12.4%, p = 0.23) and macroscopic hematuria (13.1% vs. 10.4%, p = 0.43). While there was a tendency for OP to present more often with fever, night sweat or loss of weight (11.7% vs. 4.5%), this failed to reach the level of significance (p = 0.09). The rate of lymph node metastases (YP: 3.2%, OP: 8.7%, p = 0.24) or distant metastatic disease (YP: 1.8%, OP: 5.6%, p = 0.71) at presentation did not differ significantly between both groups.

#### Histopathological features

YP showed significantly lower T stage (p = 0.03) and tumor grade (p = 0.01). While chromophobe RCC was more frequent in OP, this failed to reach the level of significance (10.7% vs. 7.1%, p = 0.32). There were no statistically significant differences in tumor location and focality, mean tumor diameter (YP: 6.5 cm, OP: 7.3 cm, p = 0.39), WHO 2002 N and M stages or UICC stage (table 2).

#### Therapy

Nephron sparing approach was chosen more often in YP (19.4% vs. 9.0%, p = 0.031). A similar proportion of patients in the two groups with nodal or distant metastasis received additional therapy (immunotherapy, tumor vaccines or conventional chemotherapy). There were no differences in mean tumor size, tumor location, performance status, ethnicity, family history of cancer, body mass index or type of treatment between YP and OP cohort.

### Predictive parameters for cancer specific survival

Mean postoperative follow-up was 52.1 months in YP and 49.4 months in OP. Table 3 shows univariate Cox regression analysis to evaluate the relationship between clinical and pathological variables and cancer specific survival. In this setting, the following prognostic factors were found: age, TNM stage, nodal status, M status, grade and clinical presentation. In addition no significant survival difference were found according to sex and histological subtypes.

**Table 3:** Univariate Cox regression analysis of factors possibly influencing cancer specific survival in patients with RCC.

		HR	95% CI	P
Age				**0.02**
	YP (≤45 years)	0.37	0.15 – 0.72	
	OP (≥75 years)	1.00	(reference)	
pTNM stage				**0.002**
	pT1+pT2	1.00	(reference)	
	pT3 +pT4	3.46	1.72 – 12.49	
Nodal status				**< 0.001**
	N0	1.00	(reference)	
	N1, N2	5.57	1.98 – 15.64	
M status				**< 0.001**
	M0	1.00	(reference)	
	M1	35.99	9.88 – 131.02	
Tumor grade				**< 0.001**
	G1+G2	1.00	(reference)	
	G3	4.01	0.73 – 21.94	
Tumor diameter				**< 0.01**
	< 7 cm	1.00	(reference)	
	≥7 cm	1.76	0.40 – 10.38	
Histological subtype				
	Clear-cell	1.00	(reference)	
	Papillary	1.43	0.46 – 4.97	0.54
	Chromophobe	1.06	0.23 – 4.78	0.92
	Other histological types	1.15	0.15 – 8.96	0.89
Sex				
	Male	1.00	(reference)	
	Female	0.61	0.24 – 1.56	0.29
Clinical presentation				**0.002**
	Asymptomatic	1.00	(reference)	
	Symptomatic	4.41	1.71 – 11.37	

bold face representing p-values < 0.05

HR = hazard ratio

CI = confidence interval

RCC = renal cell carcinoma

Young age emerged as an independent predictor of improved survival on multivariate analysis (adjusted HR 0.21, 95% CI 0.11 – 0.89, p = 0.03, table 4).

**Table 4:** Multivariate Cox regression analysis of factors possibly influencing cancer specific survival in patients with RCC.

		stepwise reverse selection		
		adjusted HR	95% CI	P

Age				**0.03**
	YP (≤45 years)	0.21	0.11 – 0.89	
	OP (≥75 years)	1.00	(reference)	
pTNM stage				**0.01**
	pT1+pT2	1.00	(reference)	
	pT3+pT4	2.82	0.74 – 10.49	
Nodal status				**0.04**
	N0	1.00	(reference)	
	N1, N2	2.37	0.95 – 5.69	
M status				**< 0.001**
	M0	1.00	(reference)	
	M1	20.61	3.32 – 127.78	
Grade				**< 0.001**
	G1+G2	1.00	(reference)	
	G3	1.65	0.18 – 15.08	
Tumor diameter				**< 0.01**
	< 7 cm	1.00	(reference)	
	≥7 cm	1.88	0.46 – 11.18	
Histological subtype				
	Clear-cell	1.00	(reference)	
	Papillary	0.78	0.14 – 3.48	0.67
	Chromophobe	1.02	0.21 – 5.75	0.90
	Other histological types	0.68	0.06 – 6.56	0.71
Sex				
	Male	1.00	(reference)	
	Female	0.52	0.38 – 2.01	0.35
Clinical presentation				
	Asymptomatic	1.00	(reference)	
	Symptomatic	0.67	0.09 – 2.33	0.49

bold face representing p-values < 0.05

HR = hazard ratio

CI = confidence interval

RCC = renal cell carcinoma

**Figure 1:**
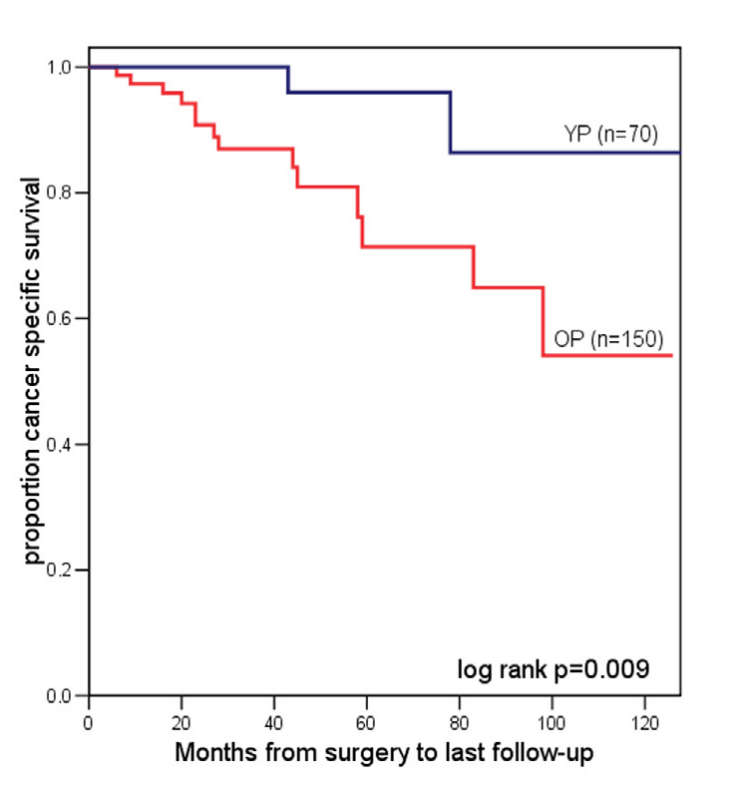
Kaplan-Meier analysis of cancer specific survival for YP (= blue line) vs. OP (= red line) with RCC.

**Figure 2:**
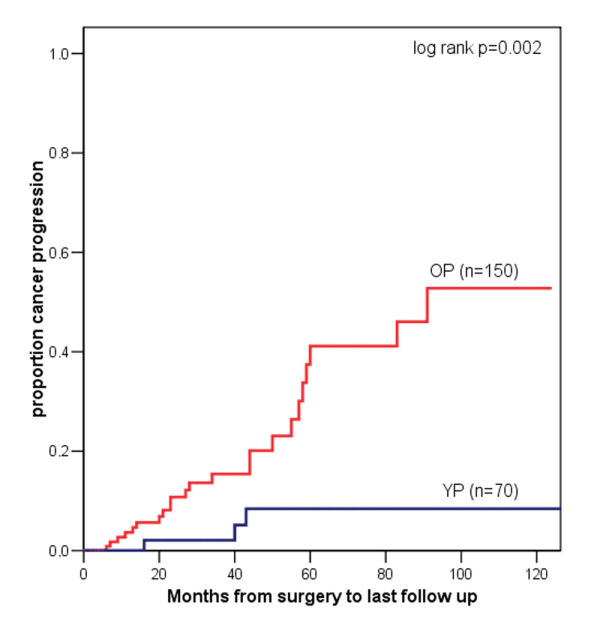
Kaplan-Meier analysis of cancer progression for YP (= blue line) vs. OP (= red line) with RCC.

Of the 70 YP three died of RCC at a mean of 3.8 years after nephrectomy. Of the 150 OP 15 died at a mean of 3.4 years following nephrectomy. One patient in YP and 23 in OP died of causes other than RCC. Kaplan-Meier survival analysis revealed a 5-year cancer specific survival rate of 95.2% for YP compared with 72.3% for OP. Log rank test did show a significantly better cancer specific survival for YP (p = 0.009; figure 1). OP more often developed systemic progress (OP: 15.3% vs. YP: 4.3%), mostly pulmonary and hepatic metastasis. The 5-year progression rate was 14.2% for YP and 41.9% for OP (log rank p = 0.002; figure 2).

## Discussion

Just 3.4% to 7.5% of all RCC patients have been reported to be young adults [7]. This is reflected in our series, as 7.2% of 968 RCC patients were aged ≤45 years. It has been suspected that young RCC is characterized by different clinicopathological and biologic character. However previous studies are somewhat contradictory and feature various methodologies. Some previous studies lack a pronounced span of age between young patients and the comparison group. While 20 years were chosen by Gillett [16], Sanchez-Ortiz chose 18 years [18], but even less has been employed [5,15,17,19]. To compare YP and OP we have determined a minimum of 30 years separating both groups. We thus hoped to increase the discrimination between young and old patients, as some overlap might occur blurring the picture. This approach seems to be backed up by epidemiological data suggesting a significantly lower incidence in patients aged up to 45 years [2].

OP were a mean 8 years above the major age of regular onset, which has been reported to be roughly 70 [2]. Thus while it has to be taken into consideration, that OP might somewhat differ from regular onset patients, the present series offers a clear separation between OP and YP. Analysis of our population confirmed the usual prognostic factors for RCC: pTNM tumor stage, tumor grade, tumor diameter, N and M stage and circumstances of renal cell carcinoma discovery. Contrary to expectations, symptoms that may be considered as substitutes for performances status were not retained as independent prognostic factors in our multivariate analysis. Moreover, recent studies considering this parameter did not show any difference according to the age at diagnosis [16,18]. Controversial data exist on the histopathological character of younger patients. In the present series YP clearly demonstrated lower T stages and lower tumor grade (table 2), while other studies only showed lower T stages in YP [6,16,18,19]. Schiff et al. compared patients younger than 40 years old to patients older than 40 years. Their data differed with the most contemporary series in that more patients in the older group presented with T1 tumors [5].

In contrast to our series a former study of wide span comparison of RCC patients < 40 years and >80 years could not figure out any differences in tumor grade [14]. A variation of distribution of histological subtypes has been reported by Gillett et al. In their to date largest series of 107 younger cases chromophobe RCC were found in 13.1% vs. 3.6% [16]. Also Rodriguez et al. found out differences in histological subtypes, OP significantly more often showed clear cell RCC than YP (91.0% vs. 69.0%, p < 0.001) [6]. In accordance with previous reports [16-19] in our series however there was an even distribution of subtypes among YP and OP. Previous authors have suggested that younger adults may have more aggressive renal tumors with a poorer prognosis than older patients [3,6]. YP tended to improved cancer specific survival in the present series. 5-year survival rate was 95.2% in YP and 72.3% in OP (p = 0.009). This finding supports previous reports [5,15], also in non comparative studies young patients showed similar survival rates [3,12,13], and is in analogy to favourable histopathology in YP. The less advanced forms of RCC found in YP may explain the better 5- year cancer specific survival. Several earlier studies [5,6] had reported comparable survival rates, despite their higher percentages of localised forms in young patients, suggesting that early-onset RCC forms might be more aggressive. However, those studies had short follow-up [17] or had small population size [5], hereby rendering interpretation of their results more difficult. In the present series, young age was an independent predictor of improved cancer specific survival. Siemer et al. found young patients to present with symptoms more often. While in their series young RCC was still related to favourable outcome, symptoms caused by malign renal masses were associated with poor prognosis [19,21]. In our series YP did not complain of symptoms more often. In synopsis the factors that determine the improved survival outcome in YP with RCC have yet to be clearly defined.

We do not believe in profoundly different tumor characters per se. This notion is supported by a significant number of young patients presenting with advanced disease and tumor related death in this group. However we suspect a reduced momentum of tumor biology in young patients. We hypothesize a better tumor control, which might be due to a superior immunology in this age group slowing the tumors in growth.

## Conclusion

To conclude RCC in younger and elder patients, as defined by a minimum of 30 years as discriminatory span, young patients present with favourable clinicopathology and an improved cancer specific 5-year survival rate after adjusting for clinical and pathological variables on multivariate analysis. These obvious differences contribute to the trend toward improve outcome in younger patients for which their young age was an independent prognostic factor. Our data refute past reports of poorer cancer specific survival in younger patients with RCC.

## Competing interests

The author(s) declare that they have no competing interests.

## Authors' contributions

**SD **participated in the design of the study, performed the statistical analysis and drafted the manuscript. **WO **prepared the data for statistical analysis and helped to draft the manuscript. **MB, KJ **and **CH **participated in the design of the study and helped to draft the manuscript. **AH **evaluated histopathological features of the study patients. **WFW **and **BW **supervised design and process of the study. All authors read and approved the final manuscript.
